# Protocol of *Birth in Brazil II: National Research on Abortion, Labor and Childbirth*

**DOI:** 10.1590/0102-311XEN036223

**Published:** 2024-04-29

**Authors:** Maria do Carmo Leal, Ana Paula Esteves-Pereira, Sônia Azevedo Bittencourt, Rosa Maria Soares Madeira Domingues, Mariza Miranda Theme, Tatiana Henriques Leite, Barbara Vasques da Silva Ayres, Márcia Leonardi Baldisserotto, Marcos Nakamura-Pereira, Maria Elisabeth Lopes Moreira, Maria Auxiliadora de Souza Mendes Gomes, Marcos Augusto Bastos Dias, Maira Libertad Soligo Takemoto, Rodolfo de Carvalho Pacagnella, Silvana Granado Nogueira da Gama

**Affiliations:** 1 Escola Nacional de Saúde Pública Sergio Arouca, Fundação Oswaldo Cruz, Rio de Janeiro, Brasil.; 2 Instituto Nacional de Infectologia Evandro Chagas, Fundação Oswaldo Cruz, Rio de Janeiro, Brasil.; 3 Universidade do Estado do Rio de Janeiro, Rio de Janeiro, Brasil.; 4 Instituto Nacional de Saúde da Mulher, da Criança e do Adolescente Fernandes Figueira, Fundação Oswaldo Cruz, Rio de Janeiro, Brasil.; 5 Levatrice Cursos, Rio de Janeiro, Brasil.; 6 Faculdade de Ciências Médicas, Universidade Estadual de Campinas, Campinas, Brasil.

**Keywords:** Guidelines as Topic, Health Surveys, Parturition, Perinatal Care, Protocolos, Inquéritos Epidemiológicos, Parto, Assistência Perinatal, Protocolos, Encuestas Epidemiológicas, Parto, Atención Perinatal

## Abstract

Brazil has made advances in obstetric care in public and private hospitals; however, weaknesses in this system still require attention. The Brazilian Ministry of Health, aware of this need, funded the second version of the *Birth in Brazil* survey. This study aimed to evaluate: prenatal, labor and birth, postpartum, and abortion care, comparing the results with those of *Birth in Brazil I*; and analyze the main determinants of perinatal morbidity and mortality; evaluate the care structure and processes of obstetrics and neonatology services in maternity hospitals; analyze the knowledge, practices, and attitudes of health professionals who provide birth and abortion care; and identify the main barriers and facilitators related to care of this nature in Brazil. With a national scope and a 2-stage probability sample: 1-hospitals and 2-women, stratified into 59 strata, 465 hospitals were selected with a total planned sample of around 24,255 women - 2,205 for abortion reasons and 22,050 for labor reasons. Data collection was conducted using six electronic instruments during hospital admission for labor or abortion, with two follow-up waves, at two and four months. In order to expand the number of cases of severe maternal morbidity, maternal and perinatal mortality, three case control studies were incorporated into *Birth in Brazil II*. The fieldwork began in November 2021 and is scheduled to end in 2023. It will allow a comparison between current labor and birth care results and those obtained in the first study and will evaluate the advances achieved in 10 years.

## Resumo

Com o passar do tempo, o Brasil vem apresentando avanços na assistência obstétrica em hospitais públicos e privados; no entanto, ainda existem pontos frágeis que necessitam de atenção. O Ministério da Saúde, ciente dessa necessidade, financiou a segunda versão da pesquisa *Nascer no Brasil*. Os objetivos gerais são: avaliar a assistência pré-natal, ao parto e nascimento, ao puerpério e ao aborto, comparando com os resultados do *Nascer no Brasil I*, e analisar os principais determinantes da morbimortalidade perinatal; avaliar a estrutura e processos assistenciais dos serviços de obstetrícia e neonatologia das maternidades; analisar os conhecimentos, atitudes e práticas de profissionais de saúde que prestam assistência ao parto e ao aborto; e identificar as principais barreiras e facilitadores para essa assistência no país. Com escopo nacional e amostra probabilística em dois estágios (1-hospitais e 2-mulheres), dividida em 59 estratos, foram selecionados 465 hospitais com total planejado de, aproximadamente, 24.255 mulheres, 2.205 por motivo de aborto e 22.050 por motivo de parto. A coleta de dados, realizada por meio de seis instrumentos eletrônicos, ocorre durante a internação hospitalar para o parto ou aborto, com duas ondas de seguimento, aos dois e quatro meses. Com o intuito de expandir o número de casos de morbidade materna grave, mortalidade materna e perinatal, três estudos caso controle foram incorporados ao *Nascer no Brasil II*. O trabalho de campo foi iniciado em novembro de 2021 com término previsto para 2023. Os resultados permitirão comparar a atenção atual ao parto e ao nascimento com a retratada no primeiro inquérito e, com isso, avaliar os avanços alcançados no decorrer desses 10 anos.

## Resumen

Aunque Brasil ha presentado avances en la atención obstétrica en hospitales públicos y privados, todavía hay puntos débiles que necesitan atención. El Ministerio de Salud, consciente de esta necesidad, financió la segunda versión de la encuesta *Nacer en Brasil*. Los objetivos generales son: evaluar la atención prenatal, el parto y el nacimiento, el puerperio y el aborto, comparando con los resultados del *Nacer en Brasil I*, y analizar los principales determinantes de la morbimortalidad perinatal; evaluar la estructura y los procesos de atención de los servicios de obstetricia y neonatología en las maternidades; analizar los conocimientos, prácticas y actitudes de los profesionales de la salud que brindan atención para el parto y el aborto; e identificar las principales barreras y facilitadores para esta atención en el país. Tiene un alcance nacional y muestra probabilística en dos etapas (1-hospitales y 2-mujeres), la cual se dividió en 59 estratos; y se seleccionaron 465 hospitales con un total planificado de aproximadamente 24.255 mujeres, de las cuales 2.205 tuvieron procedimientos por aborto y 22.050 por parto. Para la recolección de datos se aplicó seis instrumentos electrónicos, que se realizó durante la hospitalización por parto o aborto, con dos rondas de seguimiento, a los dos y cuatro meses. Con el fin de ampliar el número de casos de morbilidad materna grave, mortalidad materna y perinatal, se incorporaron tres estudios de casos y controles en *Nacer en Brasil II*. El trabajo de campo comenzó en noviembre de 2021 y finalizará en 2023. Los resultados nos permitirán evaluar la atención al parto y al nacimiento actual con lo que se retrató en la primera encuesta, de esta manera se podrá evaluar los avances alcanzados a lo largo de estos 10 años.

## Introduction

Prenatal care, labor, and birth have been the focus of several public policies in Brazil aiming to reduce maternal and child morbidity and mortality and improve the quality of women’s and children’s health care. In this context, the study *Birth in Brazil: National Survey into Labor and Birth* (*Birth in Brazil I*) was conducted in 2011-2012, coordinated by the Oswaldo Cruz Foundation (Fiocruz), with the participation of several educational and research institutions in the country.

The justification to conduct the *Birth in Brazil I* was the lack of knowledge about obstetric practices in Brazil and the increase in surgical births without clinical indication, considering the widely known impact of this procedure on the health of women and the fetus ^1^ ^,^ ^2^ ^,^ ^3^ ^,^ ^4^. The study provided the first diagnosis of labor and birth care in Brazil, exposing the magnitude of the problem, with relevant information about the characteristics of women, their gestational risk factors, access to health services, and the quality of care, labor and birth conditions, in addition to the main maternal and neonatal outcomes. It was published in thematic issues of CSP (2014, volume 30, supplement 1), and in *Reproductive Health* (2016, volume 13, supplement 3), among others.

The diagnosis in the *Birth in Brazil I* was important as it guided public policies in women’s and children’s health care, and an update of the *Birth in Brazil I* is essential for monitoring perinatal results, allowing continued monitoring of these indicators. Other countries, such as France, conduct regular perinatal studies with the same objectives because, like Brazil, they do not have an information system for detailed monitoring of perinatal care ^5^.

Despite the advances observed in obstetric care in public and private hospitals in Brazil, quality issues are still observed in areas like expansion of the appropriate use of technologies for birth and abortion care, reduction of unnecessary cesarean sections ^6^, reduction of delays in urgent and emergency cases, and improvements in the labor infrastructure of hospitals.

In this scenario, the Brazilian Ministry of Health and Fiocruz have funded the second version of the *Birth in Brazil II: National Research on Abortion, Labor and Childbirth* (*Birth in Brazil II*) for the period 2021-2023. In addition to postpartum women admitted for birth, women admitted for abortion were included in this study, given their high maternal morbidity and mortality ^7^ and because there is no nationwide study assessing their characteristics, the care provided to them in hospitals, and related complications.

In the *Birth in Brazil I*, postpartum follow-up telephone interviews were conducted to assess maternal mental health through the diagnosis of postpartum depression. A broader approach to emotional issues is used in the *Birth in Brazil II*, involving other dimensions of maternal mental health, such as post-traumatic stress disorder (associated with labor), symptoms of postpartum anxiety, quality of the mother-baby bond, and paternal mental health. Likewise, the perception of mistreatment and abuse in obstetric care, or obstetric violence, is considered in the *Birth in Brazil II*.

As with the *Birth in Brazil I*, care facilities and processes of obstetrics and neonatology services from maternity hospitals of the sample were also assessed in the *Birth in Brazil II*, in a more comprehensive manner. In addition, a knowledge, attitude, and practices survey was conducted with health professionals who provide care to women hospitalized for labor or abortion.

Three case control studies were integrated into the *Birth in Brasil II* to expand the number of rare events that could not be properly addressed in the *Birth in Brazil I*: maternal mortality; severe maternal morbidity and maternal near miss; and perinatal mortality. A qualitative study is also being conducted using telephone interviews after hospital discharge, with *Birth in Brazil II* women hospitalized for abortion. The protocols of these studies and the outline of the methodology for the investigation into the health of mothers, fathers and children during the postpartum period can be found in the Thematic Section of the CSP ^8^ ^,^ ^9^ ^,^ ^10^ ^,^ ^11^.

This study aimed to evaluate prenatal, labor and birth, postpartum, and abortion care, comparing the results with those of *Birth in Brazil I*, and analyze the main determinants of morbidity and mortality among women and newborns; evaluate the care structure and processes of obstetrics and neonatology services in maternity hospitals; analyze the knowledge, practices, and attitudes of health professionals who provide birth and abortion care; and identify the main barriers and facilitators related to this care in the country.

Box 1 shows the main questions of the *Birth in Brazil II* survey.

**Box 1:** Main questions of *Birth in Brazil II*, 2021/2023.

INVESTIGATION WITH POSTPARTUM WOMEN
Has any improvement been observed in care for pregnant women, women in labor, and newborns over this 10-year period?
Are the socioeconomic, clinical, and obstetric profiles and social and racial inequalities in prenatal, labor, and birth care associated with maternal and child outcomes?
What adverse maternal and child outcomes are associated with challenges in the access to birth care and referral services for high-risk pregnancies?
What adverse maternal and child outcomes are associated with low adequacy of prenatal care, when considering the recommendations from Stork Network program and the World Health Organization?
Which socioeconomic, clinical, and obstetric characteristics of women are associated with women’s preference for the type of birth, at the beginning and end of pregnancy?
What adverse maternal outcomes are associated with inadequate abortion and childbirth care practices?
Is the access to good practices during labor according to the recommendations of the World Health Organization associated with a lower cesarean section rate and better maternal and child outcomes?
Is the access to good practices of newborn care associated with better child outcomes?
Are failure to plan the current pregnancy and dissatisfaction with the current pregnancy associated with adverse maternal and child outcomes?
Which clinical complications that occurred in previous pregnancies, in the current pregnancy, during childbirth and in the postpartum period are associated with adverse maternal and child outcomes?
Are actions of disrespect and abuse suffered during childbirth associated with adverse maternal and child outcomes?
What characteristics of women are associated with the cesarean section rate according to Robson’s groups?
What adverse maternal and child outcomes are associated with cesarean section?
What socioeconomic, clinical, and obstetric characteristics of women are associated with vaginal birth after cesarean section?
What socioeconomic, clinical, and obstetric characteristics of women are associated with repeat cesarean section?
Is repeat cesarean section associated with adverse maternal and child outcomes?
Is the use of assisted reproduction to achieve the current pregnancy associated with adverse maternal and child outcomes?
What adverse child outcomes are associated with premature, early and late term, and post-term births?
What adverse maternal and child outcomes are associated with pre-pregnancy obesity and gestational weight gain?
What adverse maternal and child outcomes are associated with restricted intrauterine growth?
What adverse maternal and child outcomes are associated with smoking, alcohol, and illicit drug use during pregnancy?
What socioeconomic, clinical, and obstetric characteristics of women are associated with breastfeeding during hospitalization and in the first months of life?
What socioeconomic, clinical, obstetric, and care characteristics are associated with adverse late maternal (puerperium) and infant outcomes (follow-up at two and four months)?
What socioeconomic, clinical, obstetric, and care characteristics are associated with the use of health services (postpartum) and for infants (follow-up at two and four months)?
What socioeconomic, clinical, obstetric, and care characteristics are associated with unfavorable maternal mental health outcomes?
What socioeconomic, clinical, and obstetric characteristics are associated with disrespect and abuse actions during childbirth?
STUDY ABOUT CARE STRUCTURE AND PROCESSES
What adverse maternal and child outcomes are associated with inadequate structure and processes of hospital birth and abortion care services?
STUDY ABOUT KNOWLEDGE, ATTITUDE, AND PRACTICES OF HEALTHCARE PROFESSIONALS
Are knowledge, attitude, and practices of health professionals related to labor, delivery, and abortion care related to the implementation of good care practices?

## Method

The *Birth in Brazil II* is a nationwide study conducted during hospital admission for birth or abortion, with two follow-up waves at two and four months after birth. The study includes an assessment of the care structure and obstetric procedures and neonatology services in maternity hospitals and a survey on knowledge, attitudes, and practices in abortion, labor, and birth.

Data collection for the *Birth in Brazil II* started in November 2021 and is scheduled to end in 2023.

### Hospital-based study

#### Study population

The *Birth in Brazil II* survey population corresponds to the group of women hospitalized for birth (live birth or stillbirth) or abortion in hospitals with 100 or more live births per year, according to the Brazilian Information System on Live Births (SINASC). For operational reasons, women with communication issues (severe mental disorders, who do not understand Portuguese, and deaf women) and women giving birth to triplets or more were excluded. Women admitted with a diagnosis of abortion and discharged while pregnant were also excluded, as the diagnosis of abortion was not confirmed.

#### Sampling plan

A 2-stage probability sample was selected. The first stage referred to health facilities and the second referred to women.

Health facilities were classified according to information contained in the Brazilian National Registry of Health Facilities (CNES), in term of whether it is a public or private facility and, if private, whether it has beds paid for by the Brazilian Unified National Health System (SUS). Then, health facilities were classified as public, private, or mixed (private hospitals associated with the SUS).

Only hospitals with 100 or more live births per year were included in the selection for the first stage of the sample. 2,714 of the 5,710 health facilities providing labor care were eligible for the study, representing 2,861,666 (97.7%) of total 2,929,626 live births.

The hospitals were stratified according to the macroregions of Brazil (North, Northeast, Southeast, South, and Central-West), the location of the hospital (capital and municipalities in the Metropolitan Region/non-Metropolitan Region), the type of hospital (public, private, mixed), and the size of the hospital (≥ 500 live births/year and 100-499 live births/year) resulting in 60 strata. However, only 59 of the strata had hospitals with at least 100 live births/year which was the criteria for the draw (Tables 1 and 2). 

**Table 1:** Allocation of hospitals in the *Birth in Brazil II* survey in sample strata, and size, proportion of hospital sample, and sample size of postpartum women in hospitals with ≥ 500 births/year, 2022/2023.

Selection stratum	Macroregion	Metropolitan Region or non-Metropolitan Region	Hospitals	Live births	Sample of hospitals		Sample of postpartum women (n)
					n	%	
Public hospitals							
1111	North	Metropolitan Region	27	99,331	13	48.1	650
1211	North	Non-Metropolitan Region	74	78,503	11	14.9	550
	Total				24		1,200
2111	Northeast	Metropolitan Region	88	243,902	25	28.4	1,250
2211	Northeast	Non-Metropolitan Region	111	155,496	18	16.2	900
	Total				43		2,150
3111	Southeast	Metropolitan Region	129	347,385	33	25.6	1,650
3211	Southeast	Non-Metropolitan Region	43	63,429	9	20.9	450
	Total				42		2,100
4111	South	Metropolitan Region	33	65,827	10	30.3	500
4211	South	Non-Metropolitan Region	7	12,400	3	42.9	150
	Total				13		650
5111	Central-West	Metropolitan Region	25	62,419	9	36.0	450
5211	Central-West	Non-Metropolitan Region	17	17,269	4	23.5	200
	Total				13		650
Mixed hospitals *							
1121	North	Metropolitan Region	14	32,033	8	57.1	400
1221	North	Non-Metropolitan Region	13	20,720	5	38.5	250
	Total				13		650
2121	Northeast	Metropolitan Region	36	103,923	16	44.4	800
2221	Northeast	Non-Metropolitan Region	66	109,206	16	24.2	800
	Total				32		1,600
3121	Southeast	Metropolitan Region	81	165,208	22	27.2	1,100
3221	Southeast	Non-Metropolitan Region	147	196,865	26	17.7	1,300
	Total				48		2,400
4121	South	Metropolitan Region	78	135,841	19	24.4	950
4221	South	Non-Metropolitan Region	52	62,497	11	21.2	550
	Total				30		1,500
5121	Central-West	Metropolitan Region	9	28,964	7	77.8	350
5221	Central-West	Non-Metropolitan Region	29	36,706	7	24.1	350
	Total				14		700
Private hospitals							
1131	North	Metropolitan Region	10	17,934	7	70.0	350
1231	North	Non-Metropolitan Region	4	2,804	2	50.0	100
	Total				9		450
2131	Northeast	Metropolitan Region	42	76,629	23	54.8	1,150
2231	Northeast	Non-Metropolitan Region	7	5,751	3	42.9	150
	Total				26		1,300
3131	Southeast	Metropolitan Region	119	240,831	53	44.5	2,650
3231	Southeast	Non-Metropolitan Region	31	27,782	10	32.3	500
	Total				63		3,150
4131	South	Metropolitan Region	28	53,373	17	60.7	850
4231	South	Non-Metropolitan Region	6	5,595	3	50.0	150
	Total				20		1,000
5131	Central-West	Metropolitan Region	19	36,918	12	63.2	600
5231	Central-West	Non-Metropolitan Region	6	4,646	3	50.0	150
	Total				15		750
Total (hospitals ≥ 500 births/year)			1,351	2,510,187	405	30,0	20,250

* Private hospital affiliated to the Brazilian Unified National Health System.

**Table 2:** Allocation of hospitals in the *Birth in Brazil II* survey in sample strata, and size, proportion of hospital sample, and sample size of postpartum women in hospitals with 100-499 births/year, 2022/2023.

Selection stratum	Macroregion	Metropolitan Region or non-Metropolitan Region	Hospitals	Live births	Sample of hospitals		Sample of postpartum women (n)
					n	%	
Public hospitals							
1112	North	Metropolitan Region	9	1,702	2	22.2	60
1212	North	Non-Metropolitan Region	97	27,680	2	2.1	60
2112	Northeast	Metropolitan Region	42	10,474	2	4.8	60
2212	Northeast	Non-Metropolitan Region	255	54,165	2	0.8	60
3112	Southeast	Metropolitan Region	14	4,072	2	14.3	60
3212	Southeast	Non-Metropolitan Region	44	10,176	2	4.5	60
4112	South	Metropolitan Region	15	2,781	2	13.3	60
4212	South	Non-Metropolitan Region	20	4,254	2	10.0	60
5112	Central-West	Metropolitan Region	9	2,401	2	22.2	60
5212	Central-West	Non-Metropolitan Region	70	16,038	2	2.9	60
Mixed hospitals *							
1122	North	Metropolitan Region	0	0	-	-	0
1222	North	Non-Metropolitan Region	13	3,101	2	15.4	60
2122	Northeast	Metropolitan Region	6	1,783	2	33.3	60
2222	Northeast	Non-Metropolitan Region	63	16,079	2	3.2	60
3122	Southeast	Metropolitan Region	34	9,893	3	8.8	90
3222	Southeast	Non-Metropolitan Region	179	45,641	2	1.1	60
4122	South	Metropolitan Region	54	13,223	3	5.6	90
4222	South	Non-Metropolitan Region	80	21,798	2	2.5	60
5122	Central-West	Metropolitan Region	9	2,032	2	22.2	60
5222	Central-West	Non-Metropolitan Region	39	11,122	2	5.1	60
Private hospitals							
1132	North	Metropolitan Region	13	3,555	2	15.4	60
1232	North	Non-Metropolitan Region	32	7,439	2	6.3	60
2132	Northeast	Metropolitan Region	22	4,796	2	9.1	60
2232	Northeast	Non-Metropolitan Region	38	8,246	2	5.3	60
3132	Southeast	Metropolitan Region	36	10,380	2	5.6	60
3232	Southeast	Non-Metropolitan Region	61	16,125	2	3.3	60
4132	South	Metropolitan Region	27	6,577	2	7.4	60
4232	South	Non-Metropolitan Region	15	3,436	2	13.3	60
5132	Central-West	Metropolitan Region	14	3,642	2	14.3	60
5232	Central-West	Non-Metropolitan Region	53	10,914	2	3.8	60
Total (hospitals 100-499 births/year)			1,363	333,525	60	4.4	1,800
Total (hospitals ≥ 100 births/year)			2,714	2,843,712	465	17.1	22,050

* Private hospital affiliated to the Brazilian Unified National Health System.

This stratification strategy was used to ensure the selection of different types of hospital in the macroregions of the country, located both in Metropolitan Region municipalities and more remote non-Metropolitan Region municipalities.

#### Sample size and hospital allocation

Hospital allocation into strata was different for the two groups of hospitals, as described below.

#### a) Hospitals with ≥ 500 live births/year

A sample of 135 hospitals was defined for each type (public, private, mixed), totaling 405 hospitals. Hospital allocation was proportional to the number of live births in each stratum (Table 1).

The sample size was calculated using the proportion of cesarean sections of 56% in Brazil in 2019, with a significance level of 5% and power of 95%, to detect differences of 15% for the combination of hospital types and macroregions. The minimum sample size was 340 postpartum women. As the sample is grouped by hospital, a design effect of around 1.3 was used to increase the initial sample size, leading to a minimum sample size of 450 postpartum women.

Considering the combination with the lowest number of hospitals to be selected (nine hospitals for the Private-North combination), a sample of 50 postpartum women (vaginal or cesarean section birth) per hospital was defined to reach the minimum sample of 450 postpartum women (Table 1).

#### b) Hospitals with 100-499 live births/year

Hospital allocation into strata followed the same procedure, including two hospitals in each of the 30 strata, totaling 60 hospitals. A stratum could not be created due to the absence of mixed facilities with 100-499 live births/year in capitals or Metropolitan Regions in the North Region. To maintain the number of mixed hospitals with 100-499 live births/year in capitals or Metropolitan Regions, three hospitals were allocated to this stratum in the Southeast and South regions (Table 2). Because these regions are smaller, a sample of 30 postpartum women admitted for birth per hospital was defined (Table 2).

Therefore, 465 hospitals were sampled (155 public, 155 mixed, and 155 private hospitals).

#### c) All hospitals

Admissions for abortion are considered eligible for the study during the time required to identify the planned number (30 or 50) of postpartum women, without a predefined number of women admitted for abortion to be included in the study. This strategy aimed to assess the representativeness of the ratio of admissions for abortion to admissions for birth.

The total planned sample corresponded to around 24,255 women; of these, around 2,205 were admitted for abortion and 22,050 for birth (Table 2).

#### Hospital selection

#### a) Hospitals with ≥ 500 live births/year

In the first stage, hospitals were selected with the probability proportional to size (PPS) technique, which is defined by the number of live births according to SINASC. As customary in PPS selection, hospitals with a large number of live births (> 5,000/year) were included as a certainty sample and treated as selection strata for the inclusion of women. Hospitals were selected systematically, after classifying the file by stratum and number of live births in the hospital.

#### b) Hospitals with 100-499 live births/year

In the first stage, hospitals were selected via PPS technique, which is defined by the number of live births in the hospital according to SINASC. The hospitals were selected systematically, after classifying the file by stratum and number of live births.

Hospitals that did not agree to participate were replaced with the next one on the list, in the same sample stratum.

#### Selection of women

Women admitted for childbirth (vaginal or cesarean section) or abortion (spontaneous or induced, ectopic molar pregnancy) care are identified by their medical records: hospital census, admissions records and surgical procedures records. After initial identification, women are included in a single list in the chronological order of birth date for postpartum women, and the chronological order of admissions for post-abortion women.

Postpartum women are selected consecutively until the sample number is reached. The same method is applied to post-abortion women.

In hospitals with ≥ 5,000 live births/year, postpartum women are selected on alternate days, according to the childbirth date, until a sample of 50 postpartum women admitted for birth is reached. Post-abortion women are also selected on alternate days, but following the order of admission.

Losses and refusals are replaced until the expected quantity is reached in each hospital.

#### Data collection procedure

Women are approached during hospitalization, at least six hours after birth and three hours after evacuation of the uterus or surgery for ectopic pregnancy. In abortions without the need for intervention, the interview is conducted once due care has been given.

After the interval defined, women are approached in their hospital beds, whether in individual rooms or on wards. For women admitted for abortion, in addition to the face-to-face interview, an anonymous form is completed by the woman herself and deposited in a sealed box. At the end of the interview, all the women are invited to participate in two follow-up waves, two and four months after childbirth or abortion.

In every health facility, the data collection period varies according to the hospital size, the selection of study days (consecutive or alternate), and the time required for the collection of medical records of the woman and the newborn. Data collection is also performed on weekends and holidays.

#### Characteristics of data collection instruments

In the *Birth in Brazil II*, six electronic forms are used for data collection related to the woman and/or the newborn. They are completed by the research team and contain all the variables of the *Birth in Brazil I*, as well as variables regarding lifestyle, the context of the pregnancy, any mistreatment or abuse (obstetric violence), discrimination, abortion care, and maternal mental health.

The first form is used for women undergoing childbirth or abortion during hospitalization and contains variables of identification, educational level and income, obstetric history, maternal anthropometric data, physical activity, the context of the pregnancy, prenatal care, obstetric history, morbidities, and use of medication during pregnancy, intention to breastfeed, labor and an assessment of care provided to the women and the newborn. In this contact, the pregnant woman’s prenatal record card and obstetric ultrasound examinations are photographed.

The second form is completed with data from the photographed prenatal record card: clinical history, obstetric history, number of prenatal visits, blood pressure, fundal height, gestational weight, laboratory tests, nutritional supplements, medication, vaccines, diagnosis, and treatment of complications, and ultrasound data.

The third form is completed with data from prenatal medical records; hospital admission; labor, delivery, abortion and care given during birth; medication and interventions performed; maternal and neonatal morbidity; admission to an intensive care unit (ICU); and conditions for discharge of the woman and the newborn; and cause of death, if any. These data are collected after hospital discharge or up to the 42nd day of hospitalization in the case of prolonged hospitalization. Information from medical records of newborns is collected after hospital discharge or up to the 28th day of hospitalization in the case of prolonged hospitalization.

The fourth form is used for cases of abortion - except for women admitted for legal termination of pregnancy or women diagnosed with hydatidiform mole or ectopic pregnancy - as assessment of the type of abortion (spontaneous or induced) does not apply here. The form contains information on age, skin color, education, region of residence, type of hospitalization funding, type of abortion (spontaneous or induced) and abortion methods, and location and professionals involved in the case of induced abortions. It is printed, anonymous, self-completed and deposited in a sealed box.

Data is subsequently typed up by members of the research team.

The fifth form is used in the first follow-up wave, two months after birth or abortion, with questions about maternal morbidity after discharge from the hospital, use of outpatient health services, infant health, satisfaction with care received for abortion, post-traumatic stress disorder, mother-baby bond, postpartum depression, and anxiety.

The sixth form is used in the first follow-up wave, four months after birth or abortion, with questions about long COVID, breastfeeding, mistreatment and abuse (obstetric violence) in childbirth/abortion care, satisfaction with the care received during hospitalization for childbirth/abortion, and discrimination in everyday life.

The collection of information on abuse is not recommended in the hospital, as women may not report negative experiences due to the proximity of the perpetrator of abuse/mistreatment and the fact that there may be a delay in the woman understanding what has happened, particularly when abusive actions are subtle and the outcomes are positive ^12^.

The last two forms are filled in via a telephone call or an autofill link. For more details, refer to Theme-Filha et al. ^9^.

#### Variables of exposure, outcomes, and covariates

Box 2 shows the main variables of exposure, outcomes, and covariates of the *Birth in Brazil II* survey.

**Box 2:** Main variables of exposure, outcomes, and covariates of the *Birth in Brazil II* survey, 2022/2023.

SOCIODEMOGRAPHIC FACTORS AND LIFESTYLE	CONTEXT OF CURRENT PREGNANCY	MATERNAL OUTCOMES	INFANT OUTCOMES	MATERNITY HOSPITAL STRUCTURE AND PROCESSES	PRENATAL, LABOR, AND BIRTH CARE
Macroregion Location of hospital Type of hospital Birth funding Age Skin color Education Marital status Family income Paid work Religion Smoking Alcohol consumption Use of other drugs Physical activity	Sexual violence Use of assisted reproduction Use of contraceptive methods Pregnancy planning Satisfaction with pregnancy Breastfeeding intention	Type of labor start Type of delivery Type of abortion Potentially life-threatening conditions Maternal near miss Maternal death Maternal satisfaction with the hospital care provided to her Maternal satisfaction with the hospital care provided to the newborn Maternal morbidities after hospital discharge Use of primary care health services after hospital discharge Adequacy of care for postpartum women after hospital discharge Rehospitalization Mother-baby bond Post-traumatic stress disorder Postpartum depression Postpartum anxiety Self-perception of discrimination in everyday life Long COVID	Birth weight Birth length Head circumference at birth Gestational age at birth Skin-to-skin contact Breastfeeding in the first hour of life Neonatal morbidities Use of mechanical ventilation Admission to neonatal ICU Neonatal near miss Breastfeeding at hospital discharge Child morbidities after hospital discharge Use of primary care health services after hospital discharge Adequacy of newborn care after hospital discharge Rehospitalization Breastfeeding at 2 and 4 months of age	Obstetrics and neonatology Human resources Organization and work process Women’s care Maternal emergency equipment Installed capacity of beds Relationship between the hospital and the woman	Start of prenatal care Number of prenatal visits Quantitative and qualitative adequacy of prenatal care Access to childbirth care Practices in labor and birth/abortion Mistreatment in maternity hospital Quantitative and qualitative adequacy of birth/abortion care Practices with the newborn in the delivery room and during hospitalization Quantitative and qualitative adequacy of newborn care
CLINICAL INFORMATION	OBSTETRIC HISTORY	OBSTETRIC INFORMATION	EPIDEMIOLOGY	COVID-19 (ORGANIZATION TO FIGHT AGAINST IT)	
Morbidities prior to pregnancy Pre-pregnancy BMI	Parity Prior abortion/ stillbirth/neonate Prior premature/low weight Prior vaginal birth and cesarean section Interval between deliveries	Robson’s groups Morbidities occurring during pregnancy COVID-19 infection during pregnancy Hospitalization during pregnancy Weight gain during pregnancy	Equipment available Professionals comprising the team Information systems used (SIM, SINASC, SINAN) Pharmacy Availability of medications used in childbirth, postpartum, and newborn care	Human resources Supplies Data available Women and newborn care	

BMI: body mass index; ICU: intensive care unit; SIM: Brazilian Mortality Information System; SINAN: Brazilian Information System for Notificable Diseases; SINASC: Brazilian Information System on Live Births.

#### Study about the care structure and obstetrics procedures and neonatology services

#### Study population

Hospital managers and coordinators of obstetrics, neonatology, epidemiology and pharmacy services are interviewed to evaluate the care structure and obstetrics procedures and neonatology services.

#### Sampling plan

The sample includes all 465 hospitals of the *Birth in Brazil II*.

#### Data collection procedure

Professionals are interviewed face-to-face by the state research coordinator or an interviewer designated by them.

#### Form characteristics

The form consists of the following sections: characterization of the health facility (level of complexity, field of educational practice, access to blood products, laboratory tests, and ambulances); human resources (number and qualification by professional category and training activities); work organization and process in obstetrics and neonatology (availability of clinical standards and guidelines recommended by the Brazilian Ministry of Health); maternal and neonatal emergency equipment; medication; installed capacity of maternal and neonatal beds; organization of maternity wards to tackle COVID-19; and monitoring and results of care during labor, birth, and abortion. This form was developed according to current legislation ^13^ ^,^ ^14^ ^,^ ^15^ ^,^ ^16^ ^,^ ^17^ ^,^ ^18^ ^,^ ^19^ ^,^ ^20^ ^,^ ^21^ ^,^ ^22^.

### Study with health professionals who provide labor, birth, and abortion care

#### Study population

All medical professionals, nurses, psychologists, and social workers who are active during the fieldwork period and who conduct their professional activities to support women admitted for childbirth or abortion are eligible. Professionals with exclusively managerial, administrative or outpatient roles are not eligible for this study.

#### Sampling plan

The total planned sample, including all 465 hospitals of the *Birth in Brazil II*, is 4,350 health professionals.

In all 60 hospitals with less than 500 live births/year, five professionals are invited to participate: the head of the obstetrics medical staff; the head of the nursing staff, preferably the person responsible for the obstetric center; an obstetrician; a nurse; and a psychologist or social worker who supports hospitalized women. In all 405 hospitals with 500 or more live births/year, the same professionals are invited to participate, but the number increases to three physicians and three nurses, in addition to their respective heads of staff, totaling ten professionals. In both types of hospital, if a psychologist or social worker is not available, the total number of professionals is reached by interviewing more physicians and nurses.

#### Data collection procedure

Health professionals are invited to participate in the study by the *Birth in Brazil II* fieldwork team. Participation is voluntary through self-completion of an anonymous questionnaire. About 20 to 30 minutes is the time required to answer the form and the professional can choose to answer a printed or electronic form. All professionals receive an unnamed envelope containing the printed questionnaire and a link to answer it online, generated by the central coordination of the *Birth in Brazil II* survey, which will give access to an individual anonymous questionnaire that will be stored on the Fiocruz server. If the person opts to complete a printed questionnaire, this is returned in a sealed envelope to ensure anonymity, and the information is typed up by a member of the research team.

Professionals who decline the invitation to participate in the study, who do not return a completed form, who return a blank or partially completed questionnaire or who do not answer the electronic questionnaire are considered as refusals. Further professionals are contacted until the number foreseen for each type of hospital is reached, if possible by professional category, depending on availability in each hospital.

#### Characteristics of questionnaires

Three types of questionnaires are used: one for physicians, one for nurses, and one for psychologists and social workers, containing data about: (1) professional characteristics (age, sex, professional category, time since graduation, length of service at the hospital, religious affiliation, professional degree); (2) knowledge of Brazilian legislation and current care protocols for labor, birth, and early pregnancy loss; (3) attitude towards good practices for labor, birth, and pregnancy loss; Brazilian abortion law and conscientious objection to abortion; and (4) his/her practice in labor, birth, and abortion care.

### Common aspects of studies

Box 3 shows the main characteristics (data collection instruments, scope, target population, sample, ata collection period, and data collection method) of the *Birth in Brazil II* survey, including at what stage they saw the women, an evaluation of the structure of the maternity unit and an assessment of knowledge, attitudes and practices of the health professionals.

**Box 3:** Main characteristics of the *Birth in Brazil II* and the knowledge, attitudes, and practices study, 2022/2023.

	*BIRTH IN BRAZIL II*				KNOWLEDGE, ATTITUDES, AND PRACTICES STUDY
Data collection instruments	Interview with woman (1), Prenatal record card (2), and Woman-newborn’s medical records (3)	Questionnaire in sealed box (4)	Forms for 1st and 2nd follow-up interviews (5 and 6)	Form for care structure and process (7)	Questionnaire about knowledge, attitudes, and practices (8, 9 and 10)
Target population	Postpartum women and newborns and post-abortion women			Technical professional in charge	Health professionals
	Postpartum women with live births or stillbirths of ≥ 500g or ≥ 22 gestational weeks in chronological order of birth. Post-abortion women (< 500g or < 22 gestational weeks) in chronological order of hospitalization.	Post-abortion women (< 500g or < 22 gestational weeks), in chronological order of hospitalization, except those admitted for legal termination of pregnancy, hydatidiform mole or ectopic pregnancy.	Postpartum women admitted for birth or abortion interviewed at the hospital who authorized telephone contact.	Obstetrics, neonatology, epidemiology, pharmacy	Obstetrician, nurse, psychologist, social worker
Coverage	465 maternity hospitals	465 maternity hospitals	465 maternity hospitals	465 maternity hospitals	465 maternity hospitals
Sample from hospitals with 100-499 births/year	30 postpartum women + total admissions for abortion in the period until 30 postpartum women admitted for birth were obtained.	Total admissions for abortion in the period until 30 postpartum women were obtained.	Up to 30 postpartum women + abortion admissions that occurred in the period	1	5
Sample from hospitals with ≥ 500 births/year	50 postpartum women + total admissions for abortion in the period until 50 postpartum women admitted for birth were obtained.	Total admissions for abortion in the period until 30 postpartum women were obtained	Up to 50 postpartum women + abortion admissions that occurred in the period		10
Total sample estimated	Around 22,050 women	Around 2,205 women	Up to 24,255 women	465 maternity hospitals	Around 4,350 health professionals
Period of recruitment/follow-up	Period required to obtain the determined sample.	Period required to obtain the determined sample	Two and four months after childbirth or abortion	During data collection in hospitals	During data collection in hospitals
Data collection method	Face-to-face interview with the woman, photos and data extraction from the prenatal record card, extraction of data from medical records (women and newborns).	Self-completed, anonymous, deposited in a sealed box for information confidentiality.	Telephone interview or online self-completed questionnaire with the woman or related respondent.	Face-to-face interview	Online or printed self-completed anonymous questionnaire, for information confidentiality.

Note: the numbering in parentheses accounts for the total number of forms used in the survey. The study used a total of 10 forms.

All instruments are available on the *Birth in Brazil II* survey website (https://nascernobrasil.ensp.fiocruz.br/?us_portfolio=nascer-no-brasil-2).

#### Fieldwork team

For every Federative Unit (UF) in Brazil, a team was created consisting of: (a) a state coordinator and a supervisor (university professors and health professionals from state health departments), responsible for contact with health departments and maternity hospital directors to provide information on the study objectives and strategies, team selection and training, interview with the hospital manager, and fieldwork monitoring; (b) data collectors (predominantly nurses), responsible for identifying postpartum women and filling out the various data collection forms following interviews with postpartum women, medical records, photographed prenatal cards, and ultrasound reports. Also, the state team has the support of an obstetric nurse, who provides guidance and answers doubts about data collection from medical records, and a fieldwork supervisor, who is responsible for quality control and data monitoring.

#### Fieldwork team training

Training is provided to standardize the application of every data collection instrument and prepare the team for fieldwork. The training program consists of theoretical content and practical activities. The theoretical part includes a detailed description of the study, the assignments and responsibilities of data collectors, ethical aspects of research, how to use the tablet and REDCap system (https://redcap.fiocruz.br/redcap), and how to send completed forms to the Fiocruz server. Training also includes reading and application of forms, with simulation of face-to-face interviews using role play techniques, instructions on photographing prenatal cards and ultrasound reports, methods for approaching women who have undergone abortion procedures and health professionals, and simulated completion of the form with information from medical records. Practical activities are performed in the eligible healthcare facilities.

Firstly, in-person training was provided for four days, with the central team moving to the training site. It contained theoretical content and practical activities. Later, theoretical training was provided remotely by the central coordination, and practical training by the state coordination.

Strategies were developed to ensure remote training quality and participation: interview simulation in which the participant acts as the interviewer, questions about videos watched by the team, and submission of activities to the central team.

#### Pilot study

In order to assess the fieldwork logistics and the adequacy of the questionnaires in real conditions, a pilot study was conducted in the city of Rio de Janeiro, Brazil. All electronic questionnaires and information restriction and coherence programs were tested. All necessary adjustments were then made.

#### Document submission to selected hospitals

Before starting data collection, the state coordination is responsible for submitting a letter from the project coordination to the municipal administrator and the hospital director, as well as a project brief and a report from the Brazilian National Research Ethics Committee (CONEP). The hospital director is asked to sign the Informed Consent Form (ICF). At this point, a standard form is completed in order to understand the structure of the birth/abortion care provided, to allow organization of the fieldwork.

#### Quality control and data monitoring

Standardized procedures are adopted to ensure data quality and minimize systematic and random errors during data collection. The research team uses handbooks with detailed descriptions of procedures for study population selection and data collection.

Data are monitored by fieldwork supervisors to ensure the maintenance of sample representativeness and to oversee the participation rate of postpartum women and to monitor the submission of completed questionnaires to the REDCap system, hosted on the Fiocruz server. 

Unexpected situations are analyzed by coordinators to define any intervention that may be required.

#### Data analysis

Two strategies of data analysis will be used - first, a descriptive strategy, and then an analytical strategy.

First, a descriptive analysis of births and abortions will be conducted according to hospital and maternal characteristics, then indicators will be estimated for prenatal, birth/abortion, and newborn care and maternal and child outcomes (Box 2). Means and standard deviations will be calculated for continuous variables, and frequencies (percentages) for categorical variables, with respective 95% confidence intervals (95%CI). A chi-square test will be used to assess differences between proportions.

Then, to evaluate the association between the variables of exposure and outcome, unadjusted models, models adjusted for confounding factors, and models adjusted for confounding factors and mediating variables will be tested. Confounding variables will be selected using directed acyclic graphs (DAG). The main study questions (Box 1) will be tested using logistic regression, linear regression, Cox proportional hazards or generalized linear models. Complex sample analysis will be used to incorporate the effect of study design and data weighting according to the sampling plan.

#### Ethical aspects

This study was approved by CONEP (report n. 3.909.299) and local institutional review board, whenever required by selected hospitals. All precautions are adopted to ensure data secrecy and confidentiality. Data that can identify the study subjects are omitted, when submitting data to the research platform and publishing the results. After the end of the study, all study material will be stored in a database, with restricted access and supervision of the coordinating investigator.

Before every interview, the postpartum woman’s agreement is requested after reading the ICF. For minors, the assent form is used. A justification for using an invitation letter for health professionals to replace the informed consent form was submitted to CONEP, because ICF signing would allow participant identification, not ensuring the confidentiality of participant identity. The submission of a completed form was regarded as consent to participate in the study. 

## Discussion

Monitoring the processes and results of obstetric care in Brazil would alone justify the development of this second study (*Birth in Brazil II*), considering the need to reduce maternal, fetal, and infant morbidity and mortality. In the interstice between the two studies, intervention programs were implemented in the public and private systems to improve obstetric and perinatal care, including the Stork Network program ^23^ strategy and the Adequate Childbirth program ^24^, which improved the scenario presented in *Birth in Brazil I* ^6^. On the other hand, in the last five years, Brazil has seen a social and economic crisis, with important decline and disinvestment in public health policies, in addition to the COVID-19 pandemic, which impacted the access and quality of obstetric care, with consequences still unknown in maternal and child health.

In the second edition of the study, the sample representativeness increased with a higher number of hospitals (from 266 in the *Birth in Brazil I* to 465 in the *Birth in Brazil II*), mainly private hospitals (from 56 to 155), in addition to the inclusion of small hospitals (100-499 births/year). This sampling strategy increased the internal and external validity of the sample.

The inclusion of abortion in the *Birth in Brazil II* was another innovation. So far, no study has assessed abortion care in the country. Data from the *Brazilian National Abortion Survey* (PNA) conducted in 2010 and 2016 estimated a prevalence of induced abortion in the country of 15% and 13%, respectively ^25^ ^,^ ^26^. According to data from the Brazilian Hospital Information System (SIH), on average, 212,000 admissions for abortion occur annually in the country in public services ^27^. Studies conducted in the Northeast Region showed low quality of care for women admitted due to abortion complications, measured by an assessment of service structure and women’s perception of care received ^28^ ^,^ ^29^ ^,^ ^30^. Few studies with quantitative data have been developed in the country and methodological adjustments are needed. A small number of studies have been conducted outside capitals and large centers addressing the public system. Due to the illegality and stigma around abortion, inaccurate estimates occur regardless of the method and technique used ^31^. The sealed box method adopted in this study has been used in investigations about abortion and may contribute to more accurate estimates, ensuring confidentiality and protection for women. In addition the qualitative research carried out as an integral part of *Birth in Brazil II*, will allow for a deeper understanding of the healthcare needed for women undergoing abortion ^11^.

Other improvements include expansion of the sections with questions about lifestyle, intention, and satisfaction regarding pregnancy and preference for the type of birth; inclusion of (racial, social, physical, and financial) discrimination perceived in everyday life; measurement of mistreatment and abuse (obstetric violence) in its various characteristics and manifestations. According to the *Birth in Brazil I*, 44% of women reported at least one incident of physical abuse, psychological abuse, disrespectful treatment as well as a lack of privacy, information, and communication with the healthcare team, difficult access to their own and the newborn’s health status, and a loss of autonomy ^32^. In *Birth in Brazil II*, the instrument is more suitable for harvesting information about mistreatment and abuse (obstetric violence) in childbirth and abortion care, offering the possibility to explore the main consequences of these actions on the health of women and their newborns. Also, one section was included to identify potentially life-threatening conditions and manage the main obstetric complications, which had not been included in the *Birth in Brazil I*, allowing comparisons with other national and international studies.

The assessment of maternal mental health was also more comprehensive, including post-traumatic stress disorder, postpartum anxiety, mother-baby bonding, and paternal mental health ^33^. A score indicating symptoms of postpartum depression was identified in a quarter of Brazilian women in the *Birth in Brazil I* ^34^, three times higher than that reported for women in the United States ^35^.

The analysis of prenatal record card data was expanded, allowing the assessment of the suitability of various aspects of this healthcare, such as the start date of prenatal care, the number of visits, examinations, vaccines, dietary supplements, specific prophylaxis in women at high risk for pre-eclampsia, and management of complications during pregnancy. Data from the interviews with postpartum women will allow a better evaluation of the use of prenatal services, such as reasons provided by women for not accessing prenatal care or late access to this service, advice received and support to reduce/quit smoking and alcohol use during pregnancy. Advice regarding a suitable maternity hospital to avoid the need to search for this alone. Finally, data obtained from medical records will allow an evaluation of how prenatal care can effectively reduce negative outcomes, which can be prevented with actions during pregnancy, such as prevention of congenital syphilis, antepartum fetal deaths, low birth weight, anemia, and inadequate weight gain. The assessment of intrauterine growth and gestational weight gain will allow the proposal of national curves.

Given the persistence of unacceptable levels of negative maternal and perinatal indicators, combined with high coverage of hospital childbirth care, the quality of obstetric and neonatal services plays an important role in achieving improvements in maternal and child health. An assessment of the installed capacity of physical and human resources, the type of funding, and the incorporation of technology to support clinical diagnosis will allow service classification according to the degree of complexity. The type of maternity hospital organization will also help assess the provision of hospital services, the possibility of fulfilling all healthcare demands, from the simplest care needs to those requiring sophisticated technologies during abortion, labor, and birth care, as well as tackling COVID-19.

The study sampling design will allow a better understanding of the geographic distribution of physical and human resources and the identification of deficient and excess supplies and technologies in health facilities provided by the SUS and private health plans.

The evaluation of health professionals was not conducted in the *Birth in Brazil I* survey, so the implementation of labor and birth care practices was based on interviews with postpartum women and data from medical records. However, it is extremely important to evaluate the knowledge, practices, and attitudes of health professionals regarding care during childbirth and abortion and identify barriers and facilitators for the implementation of healthcare guidelines. The knowledge, attitudes and practices study integrated into the *Birth in Brazil II* will allow an assessment of the performance of professionals in different contexts across the country and a better understanding of how they and the services have experienced the changes in this field in recent years. This information will be complemented with that obtained from women, providing a more comprehensive view of the use of childbirth care services and fetal losses.

Finally, the three studies integrated into the *Birth in Brazil II* will increase the efficiency of the analysis by obtaining a higher number of cases and ensuring an adequate number of controls. Conducting these integrated studies in the same *Birth in Brazil II* hospitals with the same data collection instruments, team training, and fieldwork procedures will reduce the possibility of selection and measurement bias.

Due to its size and complexity, this study involves many challenges, but also many opportunities for innovation in research management with large-scale field data collection. Remote training mechanisms were developed to support more than 500 health professionals dedicated to data collection, using strategies to reduce face-to-face contact due to the COVID-19 pandemic, which proved to be a viable and effective solution for the fieldwork professionals distributed across the national territory. A real-time data quality control system was developed on the REDCap system, and social media communication tools were used to resolve doubts and provide instructions. Supervisors controlled the sample selection in each hospital by checking the single lists and submission of completed forms, reviewing them for completeness and quality. Decentralized coordination in every UF was essential for the study development and it included regular meetings with graduate students from the universities participating in the study for the development of theses and dissertations.

Successive health investigations with different and representative samples of the population, can be classified as trend studies. Even if the analyzed population changes, the study provides a rich source of data over time about the study base population. The *Birth in Brazil II* will allow an evaluation of the advances made in labor and birth care in public and private hospitals in order to improve the quality of abortion, labor, and birth care and reduce unnecessary cesarean sections.
